# *In situ* real-time measurement for electron spin polarization in atomic spin gyroscopes

**DOI:** 10.1016/j.isci.2025.111757

**Published:** 2025-01-06

**Authors:** Feng Li, Haoying Pang, Zhuo Wang, Wenfeng Fan, Min Zhang, Zehua Liu, Jiahang Li, Bodong Qin, Xinxiu Zhou, Xusheng Lei, Ruigang Wang

**Affiliations:** 1 Institute of Large-scale Scientific Facility and Centre for Zero Magnetic Field Science, Beihang University, Beijing 100083, China; 2 School of Instrumentation and Optoelectronics Engineering, Beihang University, Beijing 100083, China; 3 National Institute of Extremely-Weak Magnetic Field Infrastructure, Hangzhou 10587, China; 4 Hefei National Laboratory, Hefei 230088, China; 5 School of Information Science and Technology, Hangzhou Normal University, Hangzhou 311121, China

**Keywords:** Computational physics, Quantum theory, Materials characterization, Materials application

## Abstract

Atomic spin gyroscopes (ASGs) based on spin-exchange relaxation-free (SERF) co-magnetometers represent a new generation of ultra-high-precision inertial sensors. However, their long-term stability is significantly constrained by the stability of electron spin polarization. Despite its critical importance, current research lacks effective methods for *in situ* and real-time measurement of electron spin polarization. This paper addresses this gap by developing a model for pump laser propagation within the vapor cell and proposing an Euler-particle swarm optimization (PSO) algorithm to estimate the model’s unknown parameters. By utilizing artificial neural networks, we derive an output equation for electron spin polarization, using transmitted laser power and cell temperature as independent variables. Comparative experiments validate the accuracy of the proposed method, and perturbation experiments demonstrate its real-time capability. The proposed *in situ* real-time measurement method for electron spin polarization lays a solid foundation for improving closed-loop control and enhancing the long-term stability of ASGs.

## Highlights

• A model for pump laser propagation in the cell of atomic spin gyroscopes is established
• An Euler-PSO algorithm is proposed to estimate the model’s unknown parameters
• An *in situ* real-time measurement equation for electron spin polarization is developed
• The method forms the basis for closed-loop control of electron spin polarization

## Graphical abstract

**Figure:**
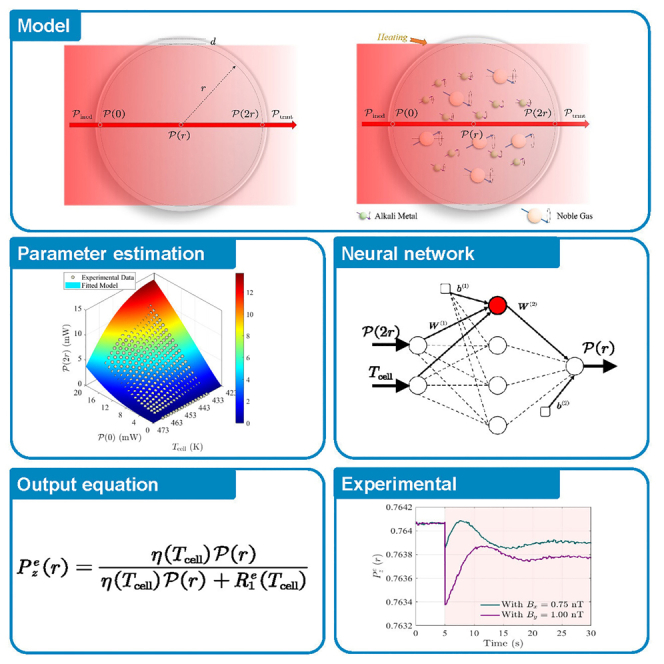

## Introduction

Quantum precision measurement instruments based on thermal atomic ensembles have become a research hotspot in recent years.^1^^,^^2^^,^^3^^,^^4^ Examples include spin-exchange relaxation-free (SERF) magnetometers,^5^^,^^6^ optical pump magnetometers (OPMs),^7^^,^^8^ atomic magnetometers based on the nonlinear magneto-optical rotation (NMOR) effect,^9^^,^^10^ and SERF co-magnetometers.^11^^,^^12^^,^^13^ Atomic spin gyroscopes (ASGs), a typical application of SERF co-magnetometers,^14^^,^^15^ offer extremely high theoretical precision for inertial measurement.

Fluctuations in electron spin polarization directly affect the output signal and the dynamic properties of the coupled atomic ensemble.^14^^,^^16^ The stability of electron spin polarization is crucial for achieving long-term stable measurements in ASGs. Therefore, closed-loop control of electron spin polarization is crucial, which requires *in situ* real-time measurement.

Li et al. measured the electron spin polarization of alkali atom Rb using optical absorption properties.^17^ However, their study did not provide a real-time measurement equation and lacked *in situ* capability. Zhao et al. calculated electron spin polarization using the slowing factor Q,^18^ but their method is offline. Wei et al. used an excitation magnetic field to analyze response signal dynamics,^19^ but this approach is also offline and less accurate. Pei et al. applied a transverse modulating magnetic field for closed-loop control.^20^ However, they did not provide a measurement equation and their method introduced interference. Cai et al. proposed a method for measuring electron spin polarization based on phase-frequency response.^21^ This method utilizes the dynamic behavior characteristics of atomic spin ensembles. However, it is not a real-time measurement method and is not suitable for closed-loop control. Overall, current research lacks effective methods for *in situ* real-time measurement of electron spin polarization.

In light of these challenges, this paper proposes a solution for *in situ* real-time measurement of electron spin polarization in ASGs. The innovative contributions of this paper are as follows.

(1) A model for the propagation of the pump laser within the vapor cell is established, and an Euler-particle swarm optimization (PSO) parameter estimation algorithm is proposed to estimate the model’s unknown parameters.
(2) The *in situ* real-time measurement equation for electron spin polarization is established based on an artificial neural network (NN), which determines the polarization at the center of the vapor cell as a function of the cell temperature and transmitted laser power.
(3) Comparative experiments validate the accuracy of the proposed method, while perturbation experiments demonstrate its real-time capability.
(4) The proposed method establishes a foundation for the closed-loop control of electron spin polarization, which has the potential to enhance the long-term stability of ASGs.

## Results

### Overview of the proposed measurement scheme

**Figure 1:**
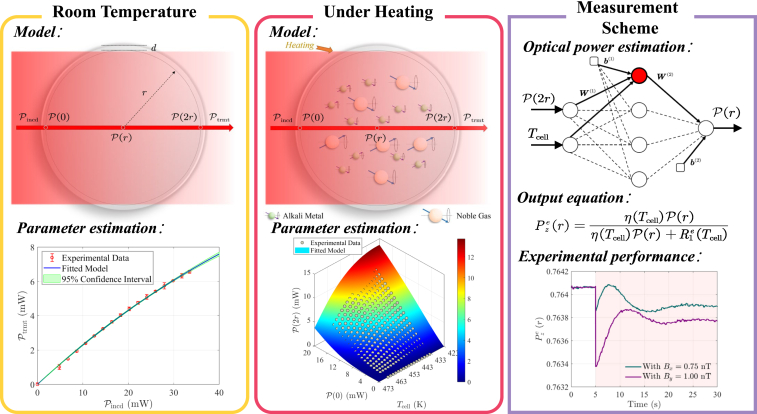
Schematic representation of the proposed *in situ* real-time measurement method

As illustrated in Figure 1, the proposed measurement scheme comprises two main components. The first component involves modeling the propagation of the pump laser within the vapor cell and experimentally determining the relevant parameters. The second component employs a neural networks (NN) to compute the electron spin polarization at the center of the vapor cell, where the probe laser traverses, based on the cell temperature and the transmitted pump laser power. Additionally, the leftmost part of the figure highlights the core components of the ASGs. Detailed results for each component will be presented in the following.

### Construction of the ASGs system

**Figure 2:**
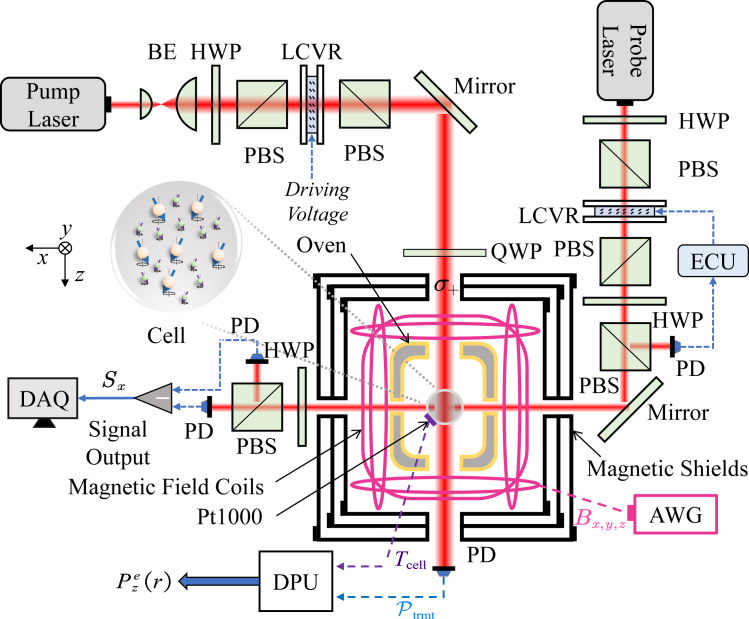
Schematic diagram of the experimental setup

Figure 2 shows a schematic of the experimental setup. The symbols used in the diagram are as follows: BE represents the beam expander, HWP stands for the half-wave plate, PBS denotes the polarization beam splitter, ECU refers to the electronic control unit, LCVR is the liquid crystal variable retarder, QWP is the quarter-wave plate, AWG refers to the arbitrary waveform generator, PD represents the photodetector, DAQ is the data acquisition system, and DPU stands for the data processing unit. The core of the experimental setup is a vapor cell with a diameter of 8 mm, containing approximately 2 atm of noble gas ^21^Ne, 20 Torr of $N_{2}$ as a quenching gas, and a mixture of alkali atoms with a density ratio of $n_{K}:n_{\text{Rb}}=1:105$ at 450 K. The cell is secured inside an oven base made of boron nitride, and its outer surface is uniformly covered by a flexible heating resistor wire to ensure indirect heating of the mixed atoms. A left-handed circularly polarized pump laser, tuned to the D1 transition of K atoms, is directed into the cell to polarize the K atoms, which further polarize the Rb atoms. Over several hours, the noble gas ^21^Ne becomes further hyperpolarized through spin-exchange interactions. The pump laser, after BE, has a spot diameter of approximately 8 mm, and its incident optical power into the cell can be adjusted by the driving voltage of the LCVR.^14^ Magnetic shielding isolates most external magnetic field interference. The remaining external magnetic fields are actively compensated by magnetic field coils driven by two arbitrary waveform generators.^22^ To maintain SERF conditions for the alkali atoms, the residual magnetic fields along the three axes are compensated to zero. The magnetic field along the longitudinal (z) axis is set to a “compensation point”, allowing the atoms to self-compensate for slow changes in the external magnetic field.^23^ A linearly polarized probe laser with a diameter of 1.5 mm is aligned along the x axis and tuned just below the D1 transition of Rb atoms to maximize the rotation signal. The probe laser power is controlled at 1 mW by the LCVR-based component. The light rotation signal caused by transverse electron spin polarization $P_{x}^{e}$ is converted into the output signal $S_{x}$ using balanced differential amplification technology.

The transmitted pump laser power is detected by a PD, while the cell temperature is measured using a platinum resistance thermometer (Pt1000). The DPU utilizes both of these measurements, along with relevant physical parameters and a trained NN model, to continuously estimate the *in situ* electron spin polarization in real-time. Using the real-time measured value as a feedback signal, a closed-loop control system can be constructed to achieve stabilization of electron spin polarization.

### Spin evolution dynamics of coupled atomic ensembles

In the ASG system, the hybrid optical pumping technique is commonly employed to achieve more homogeneous polarization of the electron spins in alkali atoms. In K-Rb-^21^Ne coupled atomic ensembles, the K atoms are directly polarized by the pump laser, while the Rb atoms are polarized through spin-exchange collisions with the K atoms. Due to the rapid spin-exchange (SE) collisions, the K and Rb atoms become effectively hybridized and can be treated as a single alkali atom.^24^ Based on this assumption, the spin evolution of both the electron spins (in alkali atoms) and the nuclear spins (in noble gas atoms) can be described by a set of partial differential equations known as the Bloch equation^16^^,^^23^^,^^25^:

(Equation 1) $$\begin{array}{cc} \frac{\partial P^{e}}{\partial t} & =\frac{\gamma^{e}}{Q}\left(\lambda M^{n}P^{n}+L+B\right)\times P^{e}-\Omega \times P^{e}-R_{\text{pu}}P^{e}\\ & +R_{\text{se}}^{en}P^{n}-R^{e}⊙P^{e}+R_{\text{pu}}S_{\text{pu}}+R_{\mathrm{pr}}S_{\mathrm{pr}}\text{,}\\ \frac{\partial P^{n}}{\partial t} & =\gamma^{n}\left(\lambda M^{e}P^{e}+B\right)\times P^{n}-\Omega \times P^{e}+R_{\text{se}}^{ne}P^{e}-R^{n}⊙P^{n}\text{.} \end{array}$$

Here, $P^{e}={\left[P_{x}^{e},P_{y}^{e},P_{z}^{e}\right]}^{T}$ denotes the electron spin polarization vector, while $P^{n}={\left[P_{x}^{n},P_{y}^{n},P_{z}^{n}\right]}^{T}$ represents the nuclear spin polarization vector. The vector $B={\left[B_{x},B_{y},B_{z}\right]}^{T}$ represents the ambient magnetic field, while $\Omega \in R^{3\times 1}$ denotes the rotation vector, which indicates the system’s angular velocity. $S_{\text{pu}}\in R^{3\times 1}$ and $S_{\mathrm{pr}}\in R^{3\times 1}$ denote the circular polarization vectors of the pump and probe lasers, respectively. $L\in R^{3\times 1}$ represents the AC-Stark shift caused by the pump and probe lasers. Q is the slowing-down factor. $\gamma^{e}$ and $\gamma^{n}$ are the gyromagnetic ratios of electrons and nuclei, respectively. $\lambda =8\pi \kappa_{0}/3$ is the geometric factor, with $\kappa_{0}$ as the enhancement factor. $M^{e}$ and $M^{n}$ denote the magnetizations corresponding to full polarization of the electrons and nuclei, respectively. $R^{e}={\left[R_{2}^{e},R_{2}^{e},R_{1}^{e}\right]}^{T}$ and $R^{n}={\left[R_{2}^{n},R_{2}^{n},R_{1}^{n}\right]}^{T}$ describe the relaxation rates of electrons and nuclei, with $R_{2}^{\cdot }$ and $R_{1}^{\cdot }$ representing transverse and longitudinal relaxation, respectively. $R_{\text{se}}^{en}$ and $R_{\text{se}}^{ne}$ are the spin-exchange rates between electrons and nuclei. $R_{\text{pu}}$ and $R_{\mathrm{pr}}$ denote the pumping rates of the pump and probe lasers. The symbol ⊙ represents the Hadamard product.

### Modeling the propagation of pump laser in the vapor cell

**Figure 3:**
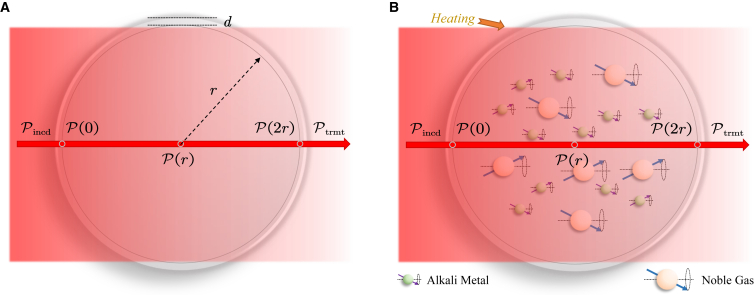
Schematic of the propagation of pump laser in the cell (A) At room temperature (≈298.15 K). (B) Under heating conditions (>423 K).

At room temperature, the alkali atoms remain approximately solid, with only inert and quenched gases present in the cell, as illustrated in Figure 3A. When heated to temperatures above 423 K, as shown in Figure 3B, the alkali atoms coexist in both liquid and gas phases, and the atomic number density of the gas phase can be described using the saturated vapor pressure formula.^26^ Compared to room temperature conditions, the pump laser power experiences additional attenuation due to the presence of alkali atoms.

#### Room temperature conditions

As illustrated in Figure 3A, the power $P_{\text{incd}}$ of the incident pump laser, after passing through a glass wall of the cell with thickness d, attenuates to:

(Equation 2) $$P\left(0\right)=P_{\text{incd}}e^{-\mu_{\text{wall}}d}$$

where $\mu_{\text{wall}}$ is the loss coefficient of the gas cell wall, and P(0) represents the initial power of the pump laser after passing through the front wall of the cell. In this loss model and the subsequent modeling, the reflection losses of the pump laser are not considered, as their contribution is relatively small.

At room temperature (approximately 298.15 K), the cell temperature is below the melting point of alkali metal atoms, meaning that the alkali atoms remain almost entirely in solid state.^27^ Therefore, the dominant effect is that the noble gas and quenching gas contribute to further attenuation of the laser power, primarily due to scattering effects.^26^ Assuming that the intensity of the gas scattering loss remains constant at different temperatures, the process can be described by the following differential equation:

(Equation 3) $$\frac{dP\left(z\right)}{dz}=-\mu_{\text{gas}}P\left(0\right)P\left(z\right)$$

where z represents the coordinate along the propagation direction of the pump laser, r is the radius of the inner wall of the cell and $\mu_{\text{gas}}$ is defined as the gas scattering loss coefficient. Therefore, the pump laser power at the end of the cell is:

(Equation 4) $$P\left(2r\right)=P\left(0\right)e^{-2P\left(0\right)\mu_{\text{gas}}r}\text{.}$$

Finally, the transmitted laser power can be expressed as:

(Equation 5) $$\begin{array}{cc} P_{\text{trmt}} & =P\left(2r\right)e^{-\mu_{\text{wall}}d}\\ & =P\left(0\right)e^{-2P\left(0\right)\mu_{\text{gas}}r}e^{-\mu_{\text{wall}}d}\\ & =P_{\text{incd}}e^{-2\left(\mu_{\text{wall}}d+P_{\text{incd}}\mu_{\text{gas}}re^{-\mu_{\text{wall}}d}\right)}\text{.} \end{array}$$

#### Under heating conditions

As shown in Figure 3B, under heating conditions (>423 K), alkali atoms exist in a state of coexistence between liquid and gas, and the atomic number density of the gas can be described using the saturated vapor pressure formula.^27^ Compared to room temperature conditions, the pump laser power will undergo further attenuation due to the presence of alkali atoms. Under heating conditions, drawing on the principles of the Lambert-Beer law,^28^ the propagation of pump laser in the cell can be described by the following differential equation:

(Equation 6) $$\frac{dP\left(z\right)}{dz}=-\mu_{\text{gas}}P\left(0\right)P\left(z\right)-n_{e}\sigma_{p}\left(\nu_{0}\right)P\left(z\right)\left[1-P_{z}^{e}\left(z\right)\right]$$

where $n_{e}$ represents the density of alkali atoms, $\sigma_{p}\left(\nu_{0}\right)$ denotes the absorption cross-section at the resonance frequency $\nu_{0}$, P(z) represents the power of the pump laser at position z and $P_{z}^{e}\left(z\right)$ is the spin polarization of alkali atoms at position z.

In the SERF atomic ensemble, spin-destruction collisions become the dominant relaxation mechanism^24^:

(Equation 7) $$R_{1}^{e}\approx \sigma_{\text{sd}}^{ee}{\bar{v}}_{ee}n_{e}+\sigma_{\text{sd}}^{en}{\bar{v}}_{en}n_{n}+\sigma_{\text{sd}}^{eN_{2}}{\bar{v}}_{eN_{2}}n_{N_{2}}$$

where $\sigma_{\text{sd}}^{ee}$ is the spin-destruction collision cross-section between alkali atoms electrons, $\sigma_{\text{sd}}^{en}$ is between alkali atoms electrons and noble gas atoms, and $\sigma_{\text{sd}}^{eN_{2}}$ is between alkali atoms electrons and quenching gas molecules. $\bar{v}=\sqrt{\frac{8k_{B}T_{\text{cell}}}{\pi \bar{m}}}$ is the relative thermal velocity between two particles, $\frac{1}{\bar{m}}=\frac{1}{m_{A}}+\frac{1}{m_{B}}$ is their reduced mass, $k_{B}$ is the Boltzmann constant, and n is the number density of the particles.

The number density of alkali atoms under high temperature can be determined by the following empirical formula:

(Equation 8) $$n_{e}\left(T_{\text{cell}}\right)=\frac{10^{21.866+k_{1}-\frac{k_{2}}{T_{\text{cell}}}}}{T_{\text{cell}}}\text{.}$$

Here, $k_{1}$ and $k_{2}$ are coefficients related to the atomic species. For the Rb atoms, the values of $k_{1}$ and $k_{2}$ are given as $k_{1}=4.312$ and $k_{2}=4040$,^26^ respectively.

The pumping rate $R_{\text{pu}}\left(z\right)$ of alkali atoms electrons takes the following form:

(Equation 9) $$R_{\text{pu}}\left(z\right)=\sigma_{p}\left(\nu_{0}\right)\Phi \left(z\right)=\frac{2cr_{e}f}{\Gamma h\nu_{0}A}P\left(z\right)$$

where $\Phi \left(z\right)=\frac{P\left(z\right)}{h\nu_{0}A}$ is the flux of photons per unit area per unit time, c is the speed of light, $r_{e}$ is the electron radius, f is the oscillation strength, Γ is the pressure broadening, h is the Planck constant, and A is the area of the pump laser spot. Then η is defined as the pumping rate corresponding to the power of pump laser per watt (sensed by the atoms), with units of $s^{-1}W^{-1}$. Finally, the aforementioned differential Equation 6 can be rewritten as:

(Equation 10) $$\begin{array}{cc} \frac{dP\left(z\right)}{dz} & =-\mu_{\text{gas}}P\left(0\right)P\left(z\right)-n_{e}\frac{2cr_{e}f}{\Gamma }P\left(z\right)\left(1-\frac{R_{\text{pu}}\left(z\right)}{R_{\text{pu}}\left(z\right)+R_{1}^{e}}\right)\\ & =-\mu_{\text{gas}}P\left(0\right)P\left(z\right)-n_{e}Ah\nu_{0}\eta P\left(z\right)\left(\frac{R_{1}^{e}}{\eta P\left(z\right)+R_{1}^{e}}\right)\text{.} \end{array}$$

With this, the propagation model of the pump laser in the cell is established. Due to the presence of the first term in Equation 10, fast calculations using the Lambert-W function, as traditionally employed, are not feasible. In other words, the unknown parameters $R_{1}^{e}$ and η cannot be determined through data fitting as would be possible with algebraic equations.

### Parameter estimation based on the Euler-PSO algorithm

#### Theoretical approach

To estimate the unknown parameters $R_{1}^{e}$ and η in Equation 10, this paper proposes an Euler-PSO parameter estimation algorithm.

Considering the approximate differential in the following form, which represents the first-order Euler method^29^:

(Equation 11) $$\frac{dP\left(z\right)}{dz}\approx \frac{P\left(\;\Delta z\left(k+1\right)\right)-P\left(\Delta zk\right)}{\Delta z}$$

where k represents the iteration index and Δz denotes the step size in the discretized spatial domain. This approximation holds when Δz is sufficiently small. Thus, (10) can be reformulated into the following discrete difference equation:

(Equation 12) $$P\left(\;\Delta z\left(k+1\right)\right)=\Delta z\left[\mu_{\text{gas}}P\left(0\right)P\left(\;\Delta zk\right)-n_{e}Ah\nu_{0}\eta P\left(\;\Delta zk\right)\left(\frac{R_{1}^{e}}{\eta P\left(\Delta zk\right)+R_{1}^{e}}\right)\right]+P\left(\;\Delta zk\right)\text{.}$$

PSO is a stochastic optimization algorithm inspired by the social behavior of bird flocks and fish schools.^30^^,^^31^ It iteratively searches for the optimal solution by simulating the collective movement of particles in a multidimensional search space. Unlike conventional optimization techniques, such as gradient descent or Newton’s method, which typically rely on gradient information, PSO does not require gradient-based information and is well-suited for optimizing non-linear, high-dimensional problems. Its ability to explore large solution spaces and avoid local minima makes it particularly effective for parameter fitting in model (12).

The PSO algorithm relies on two key equations. First, the velocity update equation adjusts each particle’s velocity:

(Equation 13) $$v_{i}^{j+1}=\omega^{j}v_{i}^{j}+c_{1}r_{1}\left(p_{i}-x_{i}^{j}\right)+c_{2}r_{2}\left(g-x_{i}^{j}\right)\text{.}$$

Second, the position update equation updates each particle’s position based on its velocity:

(Equation 14) $$x_{i}^{j+1}=x_{i}^{j}+v_{i}^{j+1}\text{.}$$

In these equations, $v_{i}^{j}=\left[v_{i1}^{j},v_{i2}^{j},\ldots ,v_{iD}^{j}\right]$ represents the velocity of particle i at iteration j, and $x_{i}^{j}=\left[x_{i1}^{j},x_{i2}^{j},\ldots ,x_{iD}^{j}\right]$ denotes the position of particle i at iteration j. The term $p_{i}=\left[p_{i1},p_{i2},\ldots ,p_{iD}\right]$ refers to the historical best position of particle i, while $g=\left[g_{1},g_{2},\ldots ,g_{D}\right]$ is the global best position found by the swarm. $c_{1}$ and $c_{2}$ are the cognitive and social acceleration coefficients, respectively. $c_{1}$ dictates the weight that a particle assigns to its own best-known position $p_{i}$, promoting exploration based on the particle’s individual experience. $c_{2}$ determines the weight that a particle assigns to the global best position g, encouraging convergence toward the best solution found by the swarm. Together, these coefficients balance the particle’s tendency to explore new areas and exploit known good solutions. $r_{1}$ and $r_{2}$ are random scalars uniformly distributed in the range [0, 1], introducing stochasticity into the velocity update to help diversify the search and avoid local minima. The inertia weight $\omega^{j}$ is crucial for balancing exploration and exploitation, and it is adapted over the iterations as follows:

(Equation 15) $$\omega^{j}=\frac{\omega_{\max}-\omega_{\min}}{1+e^{-\xi j}}+\omega_{\min}$$

where $\omega^{j}$ represents the inertia weight at the j-th iteration. $\omega_{\max}$ and $\omega_{\min}$ denote the maximum and minimum values of the inertia weight, respectively, and ξ is the parameter that controls the rate of change of the inertia weight over the iterations, typically ranging from 0.01 to 0.1.

The fitness function is a crucial component of the PSO algorithm, as it assesses each particle’s position by comparing computed values with experimental data. Specifically, it evaluates how closely the computed P(2r) matches the experimental data $P_{\exp}\left(2r\right)$, usually by calculating the Euclidean distance. The fitness function is defined as follows:

(Equation 16) $$J\left(x_{i}^{j+1}\right)={\left\|P_{\exp}\left(2r\right)-P\left(2r\right)\right\|}_{2}\text{.}$$

This fitness measure directs particles toward areas in the search space where computed values closely match the experimental data, thereby guiding the optimization process toward the optimal solution.

In summary, we present the Euler-PSO parameter estimation algorithm for determining the parameters of coupled spin ensembles that interact with the pump laser. The pseudocode of this algorithm is provided in Algorithm 1.

Algorithm 1 **Euler-PSO parameter estimation algorithm**

1: **Input:** Initial guess for $\left[\eta ,R_{1}^{e}\right]$, population size N, maximum iterations M, step size Δz

2: **Output:** Estimated parameters $\left[\eta ,R_{1}^{e}\right]$

3: Initialize particle positions $\left[\eta_{i},R_{1i}^{e}\right]$ and velocities $v_{i}$ for i=1,2,…,N

4: Initialize $p_{i}$ as the best known position of particle i

5: Initialize g as the best known position of the swarm

6: Load the experimental data $P_{\exp}\left(2r\right)$

7: Initialize $\omega_{\max},\omega_{\min},\xi$ for adaptive inertia weight

8: Set $\omega^{0}=\omega_{\max}$ ▹ Initialize inertia weight

9: Set j=0

10: **while** j≤M **do**

11: **for** each particle i **do**

12: Update velocity using Equation 13

13: Update position using Equation 14

14: Initialize $P\left(0\right)=P_{0}$ and set z=0

15: Set k=0

16: **while** Δz(k+1)≤2r **do**

17: Compute $\hat{P}\left(\Delta z\left(k+1\right)\right)$ using Equation 12

18: k=k+1

19: **end while**.

20: Evaluate the fitness $J\left(x_{i}^{j+1}\right)$ by comparing P(2r) with $P_{\exp}\left(2r\right)$ using Equation 16

21: **if** fitness $J\left(x_{i}^{j+1}\right)$ better than fitness $J\left(p_{i}\right)$ **then**

22: Update $p_{i}=x_{i}^{j+1}$

23: **end if**.

24: **if** fitness $J\left(x_{i}^{j+1}\right)$ better than fitness J(g) **then**

25: Update $g=x_{i}^{j+1}$

26: **end if**.

27: **end for**.

28: j=j+1 ▹ Update iteration counter

29: Update the inertia weight $\omega^{j}$ using the adaptive strategy (15)

30: **end while**.

31: **return** g as the best found parameters $\left[\eta ,R_{1}^{e}\right]$

#### Experimental procedure and results

**Figure 4:**
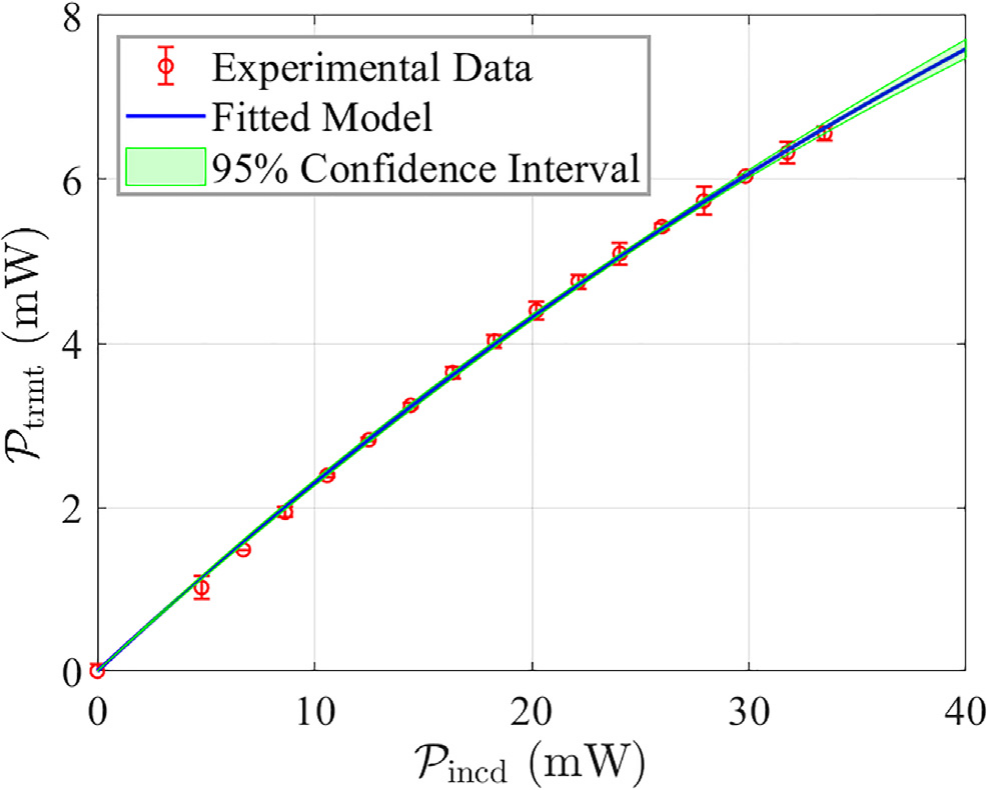
Fitting results with data obtained from seven repeated experiments under identical conditions

Under room temperature conditions, the incident pump laser power $P_{\text{incd}}$ is varied while recording the corresponding transmitted power $P_{\text{trmt}}$. The experimental data are then fitted using the model in Equation 5, and the results are shown in Figure 4. The figure indicates that the experimental data align closely with the fitted model, validating the accuracy of the fit and allowing for precise determination of the parameters $\mu_{\text{wall}}$ and $\mu_{\text{gas}}$.

**Figure 5:**
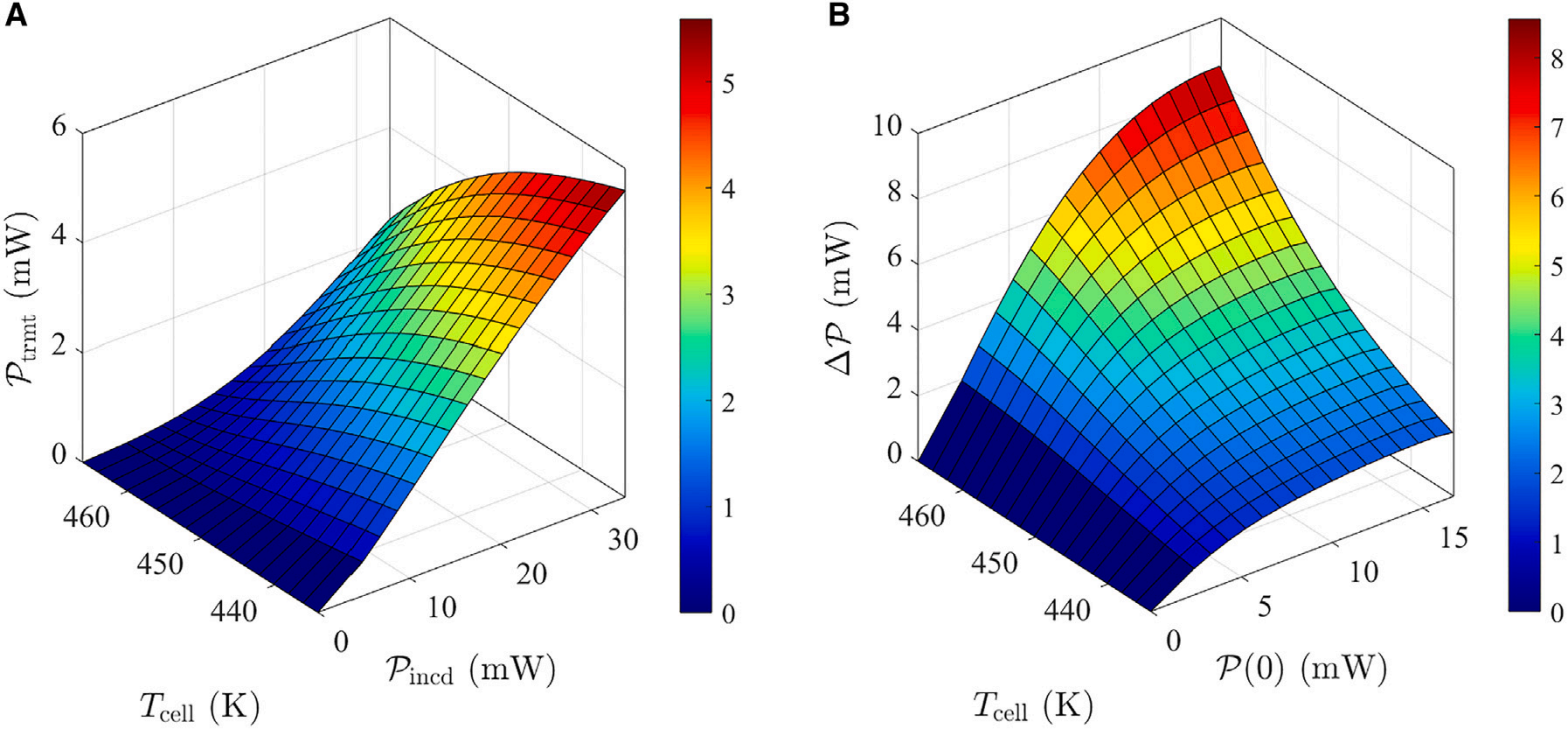
Experimental results (A) Variation in transmitted laser power $P_{\text{trmt}}$ with different cell temperatures $T_{\text{cell}}$ and incident laser power $P_{\text{incd}}$. (B) Optical power loss ΔP under different $T_{\text{cell}}$ and P(0) values.

Subsequently, under heating conditions, the cell temperature $T_{\text{cell}}$ and incident laser power $P_{\text{incd}}$ are varied simultaneously, while recording the transmitted laser power $P_{\text{trmt}}$. The experimental results shown in Figure 5A are obtained accordingly.

Then, the optical power loss caused by atoms inside the cell at high temperatures can be defined as:

(Equation 17) $$\Delta P=P_{R}\left(2r\right)-P\left(2r\right)\text{.}$$

Here, $P_{R}\left(2r\right)$ and P(2r) represent the power at the end of the cell under room temperature and heating conditions, respectively, calculated by model (5). The optical power loss ΔP under different P(0) values (calculated by model (2)) and varying temperatures $T_{\text{cell}}$ are plotted in Figure 5B. It can be observed that as P(0) increases, the optical power loss ΔP gradually increases and then converges. The analysis suggests that this is due to the gradual saturation of the alkali atoms polarization, leading to a stabilization in the absorption intensity of the pump laser.

**Figure 6:**
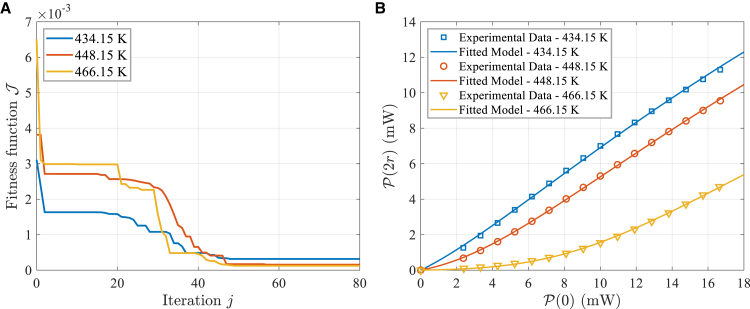
Algorithm running results (A) The convergence process of the fitness function J. (B) The fitting results.

Subsequently, the data under heating conditions are used to estimate the unknown parameters η and $R_{1}^{e}$ of model (10) using Algorithm 1. Exemplarily, the convergence process of the fitness function and the fitting results at three different temperatures during the execution of the algorithm are shown in Figure 6. The experimental data align well with the fitted model, confirming the reliability of the estimated values for η and $R_{1}^{e}$.

Based on Equation 7, we can describe the relationship between $R_{1}^{e}$ and the vapor cell temperature $T_{\text{cell}}$ using the following model:

(Equation 18) $$R_{1}^{e}\left(T_{\text{cell}}\right)=q_{v}\sqrt{\left(T_{\text{cell}}\right)}+q_{nv}\sqrt{\left(T_{\text{cell}}\right)}\frac{10^{21.866+k_{1}-\frac{k_{2}}{T}}}{T_{\text{cell}}}+q_{0}$$

where $q_{v}$, $q_{nv}$, and $q_{0}$ are the parameters to be fitted. Specifically, $q_{v}$ is the coefficient that relates to the square root of the cell temperature, $q_{nv}$ is the coefficient that depends on both the square root of the cell temperature and the atomic number density of the alkali atoms, and $q_{0}$ is the bias coefficient.

**Figure 7:**
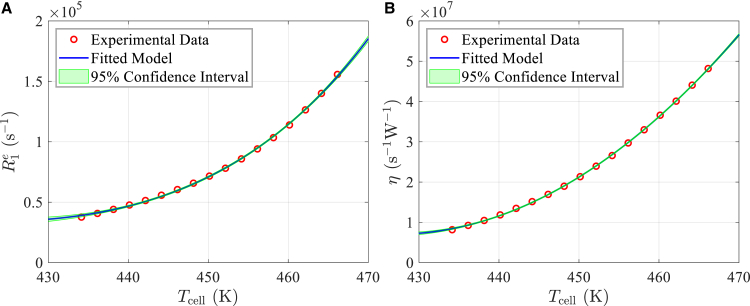
Fitting results (A) The relationship between $R_{1}^{e}$ and the vapor temperature $T_{\text{cell}}$. (B) The relationship between η and the cell temperature $T_{\text{cell}}$.

The relationship between parameter η and the cell temperature $T_{\text{cell}}$ is complex, as it is influenced by various factors such as atomic density, atomic motion, and the interaction between the pump laser and the alkali atoms. Due to these interdependent effects, deriving a precise physical model for η as a function of $T_{\text{cell}}$ is challenging. Therefore, a quadratic polynomial is used as an approximation to describe this relationship:

(Equation 19) $$\eta \left(T_{\text{cell}}\right)=w_{0}{\left(T_{\text{cell}}\right)}^{2}+w_{1}\left(T_{\text{cell}}\right)+w_{3}$$

where $w_{0}$, $w_{1}$ and $w_{3}$ are the parameters to be fitted. The values of η and $R_{1}^{e}$ obtained from running Algorithm 1 are fitted to these two models, resulting in the outcomes shown in Figure 7. Then, the unknown parameters in Equations 18 and 19 are determined.

**Figure 8:**
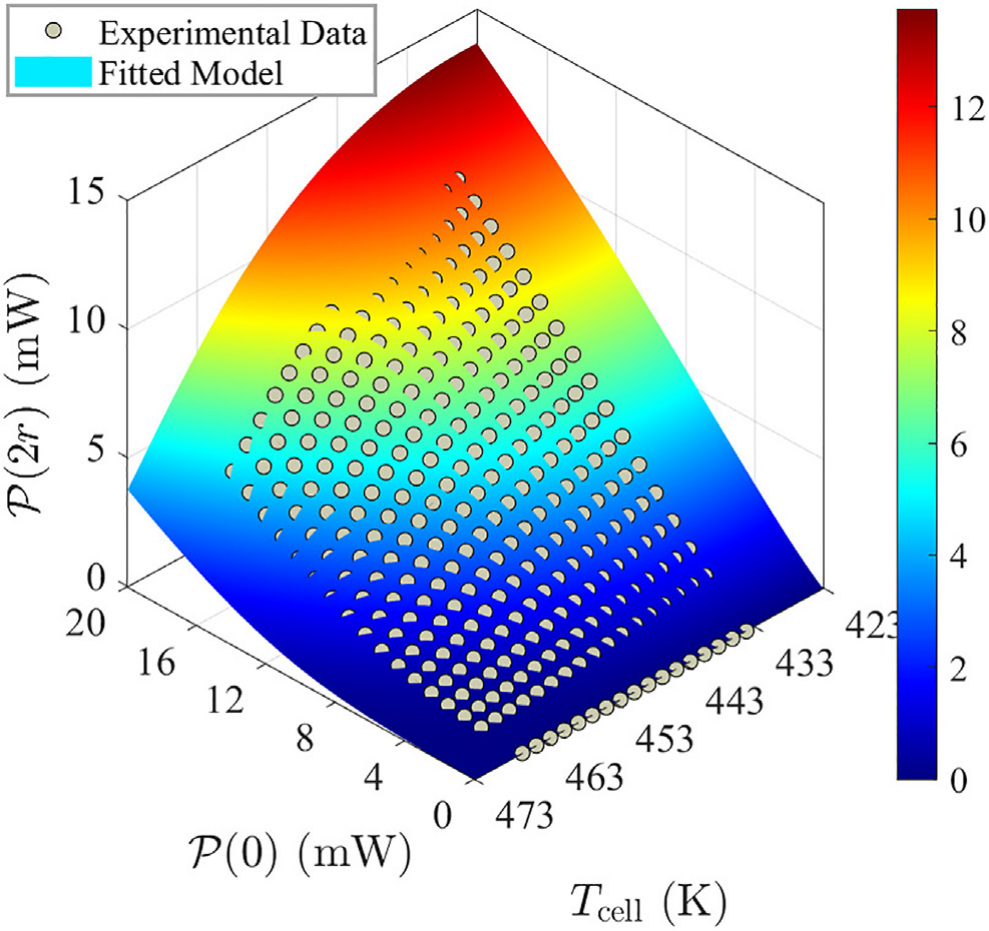
Experimental data (scatter points) and the fitted model (surface plot) for the pump laser power P(2r) as a function of the pump laser power P(0) and the cell temperature $T_{\text{cell}}$

Finally, by combining Equations 12, 18, and 19, Figure 8 illustrates the match between the established model and the experimental data, demonstrating the model’s accuracy.

### *In situ* real-time measurement of electron spin polarization

After the propagation model of the pump laser within the cell is established, the next step is to determine the electron spin polarization $P_{z}^{e}\left(r\right)$ at the center of the vapor cell using only the transmitted laser power $P_{\text{trmt}}$ and the cell temperature $T_{\text{cell}}$. Neural networks (NNs) excel at fitting nonlinear functions due to their powerful representational capacity and automatic feature extraction, which enable efficient and accurate modeling of complex data patterns.^32^^,^^33^^,^^34^ This study utilizes a single-hidden-layer feedforward NN to achieve *in situ* real-time measurement of the electron spin polarization $P_{z}^{e}\left(r\right)$. First, using the previously established model, a large dataset was generated for $T_{\text{cell}}$, P(2r), and P(r). Subsequently, $T_{\text{cell}}$ and P(2r) were used as inputs to train the NN, while P(r) served as the output. The iteratively trained NN, combined with P(2r) and $T_{\text{cell}}$, allows for the determination of the laser power P(r) at the center of the cell. Finally, the output equation for $P_{z}^{e}\left(r\right)$ is derived, which is a function of the transmitted laser power $P_{\text{trmt}}$ and the cell temperature $T_{\text{cell}}$.

**Figure 9:**
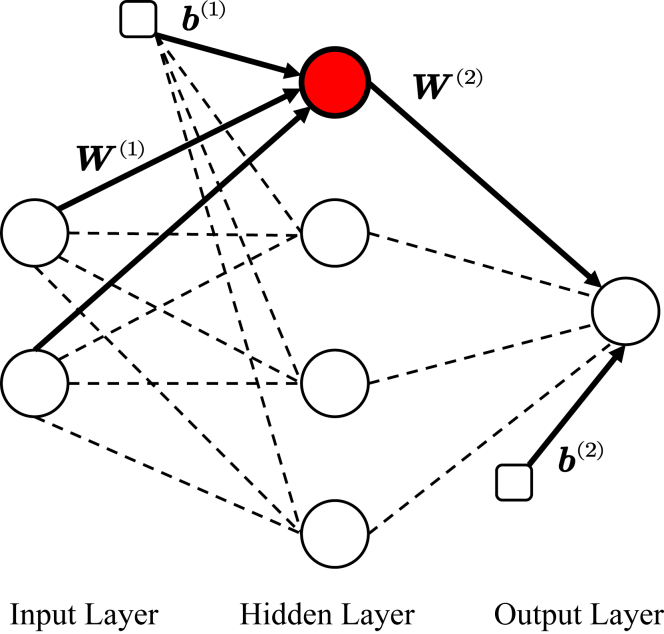
Schematic diagram of the single-hidden-layer feedforward neural network

The output of the single-hidden-layer feedforward NN as shown in Figure 9 can be described by the following formula^32^^,^^33^:

(Equation 20) $$y_{\text{NN}}=W^{\left(2\right)}\left(\frac{2}{1+\exp\left(-2\left(W^{\left(1\right)}x+b^{\left(1\right)}\right)\right)}-1\right)+b^{\left(2\right)}$$

where $y_{\text{NN}}$ is the output vector of the NN, x is the input vector to the NN, $W^{\left(1\right)}$ is the weight matrix connecting the input layer to the hidden layer, $b^{\left(1\right)}$ is the bias vector for the hidden layer, $W^{\left(2\right)}$ is the weight matrix connecting the hidden layer to the output layer, and $b^{\left(2\right)}$ is the bias vector for the output layer. The term inside the activation function, $\frac{2}{1+\exp\left(-2\left(W^{\left(1\right)}x+b^{\left(1\right)}\right)\right)}-1$, represents the hyperbolic tangent (tanh) activation function applied element-wise to the linear combination of the input vector x, the hidden layer weight matrix $W^{\left(1\right)}$, and the hidden layer bias vector $b^{\left(1\right)}$.

First, utilizing the model (12) established earlier, a substantial dataset comprising the cell temperature $T_{\text{cell}}$, the laser power at the end of the cell P(2r), and the power at the center of the cell P(r) is generated. Subsequently, $T_{\text{cell}}$ and P(2r) are employed as inputs for training the NN, while P(r) served as the output. The training process adjusts the weights $W^{\left(1\right)}$, $W^{\left(2\right)}$, and biases $b^{\left(1\right)}$, $b^{\left(2\right)}$ of the NN to minimize the error between the predicted outputs $y_{\text{NN}}$ and the actual values of P(r), ensuring the model captures the relationship between the input parameters and the desired output accurately.

**Figure 10:**
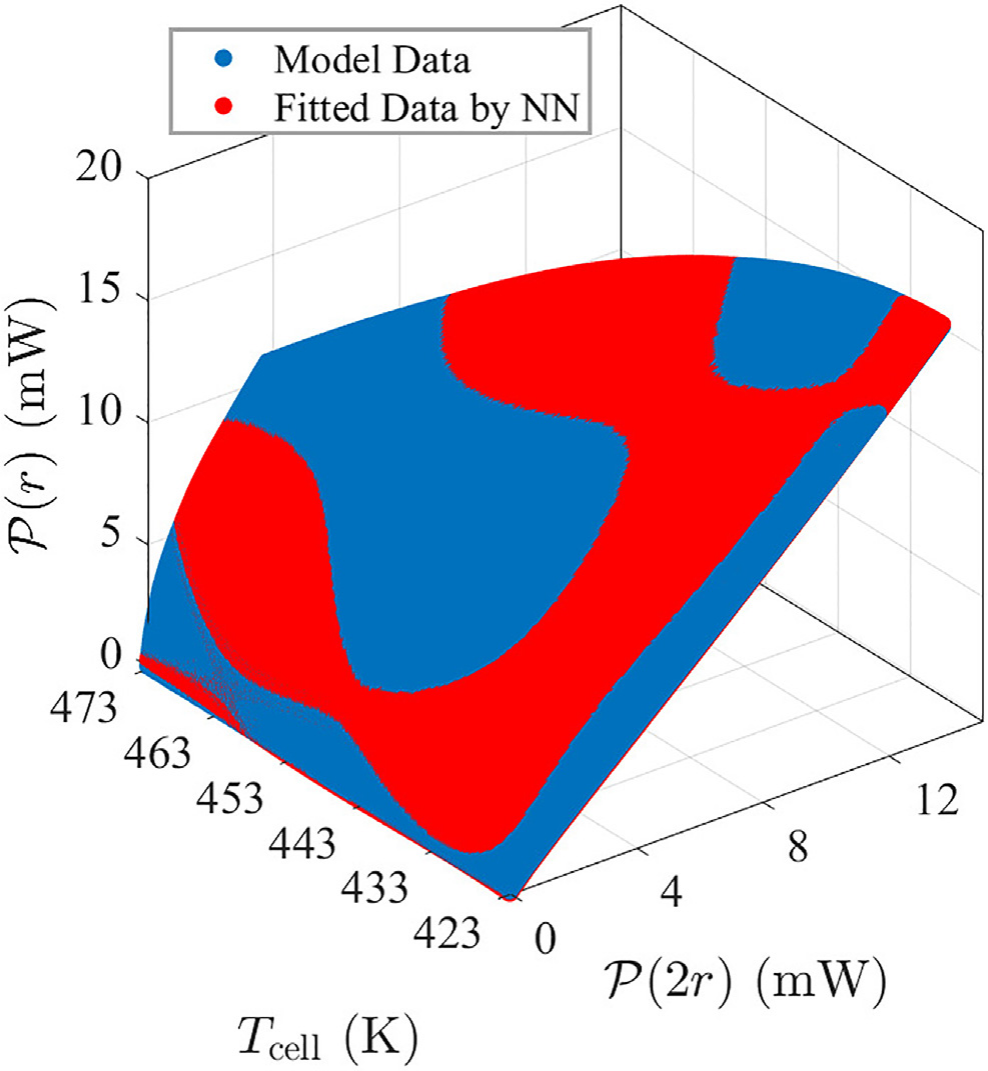
The training results of the neural network

Figure 10 illustrates the fitting of the trained NN to the data generated by the previously established model. The scatterplot shows the original model data (blue filled circles) and the data fitted by the NN (red filled circles) as a function of P(2r) and $T_{\text{cell}}$. To quantify the model’s accuracy, the coefficient of determination $\left(R^{2}\right)$ is introduced^35^:

(Equation 21) $$R^{2}=1-\frac{\sum_{i=1}^{n}{\left(y_{i}-{\hat{y}}_{i}\right)}^{2}}{\sum_{i=1}^{n}{\left(y_{i}-\bar{y}\right)}^{2}}$$

where $y_{i}$ represents the actual value (the model data), ${\hat{y}}_{i}$ denotes the estimated value (the fitted data), $\bar{y}$ is the mean of the actual values, and n is the total number of data points. With $R^{2}=0.999$ after calculation, which is nearly 1, it can be concluded that the constructed NN model achieves a high degree of accuracy.

Finally, the electron spin polarization at the center of the cell can be represented as:

(Equation 22) $$P_{z}^{e}\left(r\right)=\frac{R_{\text{pu}}\left(r\right)}{R_{\text{pu}}\left(r\right)+R_{1}^{e}\left(T_{\text{cell}}\right)}=\frac{\eta \left(T_{\text{cell}}\right)P\left(r\right)}{\eta \left(T_{\text{cell}}\right)P\left(r\right)+R_{1}^{e}\left(T_{\text{cell}}\right)}$$

where

(Equation 23) $$P\left(r\right)=W^{\left(2\right)}\left(\frac{2}{1+\exp\left(-2\left(W^{\left(1\right)}{\left[P_{\text{trmt}}e^{\mu_{\text{wall}}d},T_{\text{cell}}\right]}^{T}+b^{\left(1\right)}\right)\right)}-1\right)+b^{\left(2\right)}$$

with $W^{\left(1\right)}\in R^{4\times 2}$ , $W^{\left(2\right)}\in R^{1\times 4}$, $b^{\left(1\right)}\in R^{4\times 1}$, and $b^{\left(2\right)}\in R^{1\times 1}$. According to this equation, the electron spin polarization $P_{z}^{e}\left(r\right)$ at the center of the vapor cell is determined using the transmitted laser power $P_{\text{trmt}}$ and the cell temperature $T_{\text{cell}}$.

**Figure 11:**
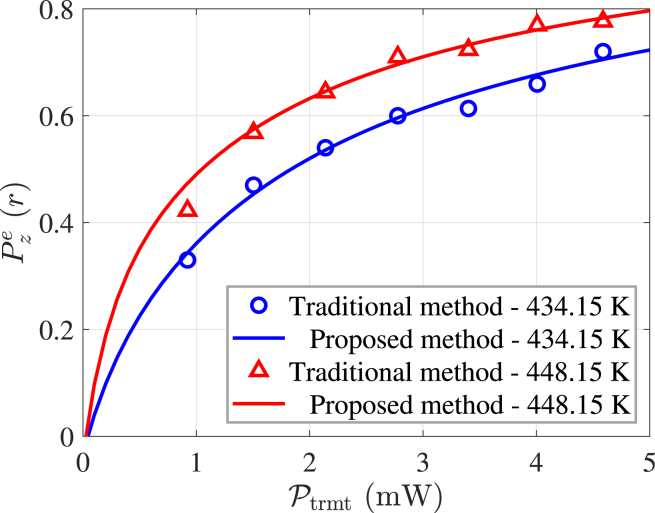
Comparison with the measurement results of the offline method

To validate the accuracy of the proposed measurement method, we compare it with a traditional offline measurement technique.^18^ The experimental results are illustrated in Figure 11. Measurements obtained using the proposed method show good agreement with those from the traditional method. Additionally, the proposed method provides the benefits of real-time and rapid measurement capabilities.

**Figure 12:**
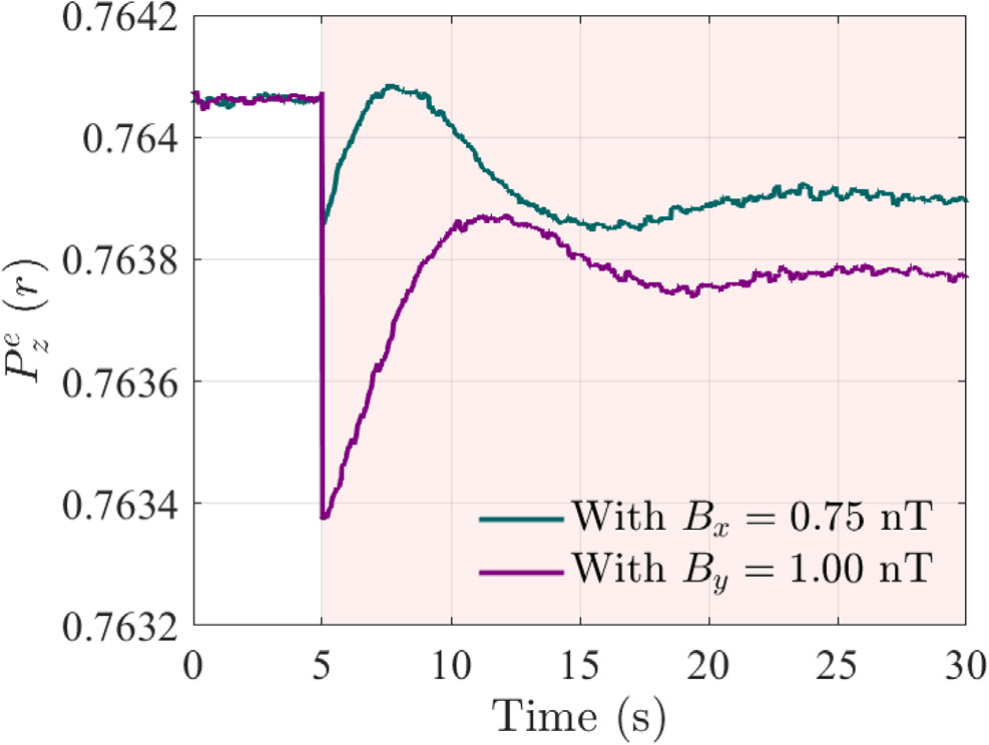
Real-time measurement results

To verify the real-time capability of the proposed method, two types of magnetic field perturbations are applied to the ASGs: $B_{x}$ = 0.75 nT along the x axis and the $B_{y}$ = 1.00 nT along the y axis. The time evolution of the electron spin polarization $P_{z}^{e}\left(r\right)$ is shown in Figure 12. The measurement results show that after applying the perturbation field, the value of $P_{z}^{e}\left(r\right)$ experienced a transient change before stabilizing.

## Discussion

This paper enables *in situ* real-time measurement of electron spin polarization. First, a model for pump laser propagation within the vapor cell is developed, and an Euler-PSO parameter estimation algorithm is introduced to estimate the model’s unknown parameters. Next, the output equation for electron spin polarization at the center of the cell is derived using a NN. The experimental results show that the developed model closely matches the data, confirming its accuracy. Finally, comparative experiments confirm the effectiveness of the proposed *in situ* measurement method, while perturbation excitation experiments demonstrate its real-time capability.

Previous studies have faced challenges due to the lack of direct measurement methods, leading to the stability of electron spin polarization in ASG systems being indirectly achieved through stabilizing the incident pump laser branch. This method has resulted in suboptimal performance. In contrast, the output equation for electron spin polarization derived in this study can be directly used to design controllers. The proposed *in situ* real-time measurement method provides a foundation for closed-loop control of electron spin polarization, thereby enhancing the stability of ASG output signals.

### Limitations of the study

In this study, we neglect the laser reflection loss, as its contribution is relatively small. Additionally, we do not model the laser power in the direction perpendicular to the laser propagation.

## Resource availability

### Lead contact

Requests for further information and resources should be directed to and will be fulfilled by the lead contact, Haoying Pang (panghaoying@buaa.edu.cn).

### Materials availability

This study did not generate new materials.

### Data and code availability

• The lead contact will provide access to the data presented in this study upon request.
• The lead contact will share the code used in this paper upon request.
• Any additional information required to reanalyze the data reported in this paper is available from the lead contact upon request.

## Acknowledgments

This work was supported in part by the Innovation Program for Quantum Science and Technology (grant nos. 2021ZD0300400 and 2021ZD0300402), the Postdoctoral Fellowship Program of CPSF (grant no. GZC20233385), the National Natural Science of China for Distinguished Young Scholars (grant no. 61925301), the 10.13039/501100001809National Natural Science Foundation of China (grant nos. 61673041, 62122009, 62103026, and 62303035), the 10.13039/501100015956Key Area Research and Development Program of Guangdong Province (grant no. 2021B0101410005), and the 10.13039/501100002858China Postdoctoral Science Foundation (grant no. 2024T171116).

## Author contributions

F.L., H.P., Z.W., W.F., M.Z., X.Z., and X.L. proposed this study. F.L., Z.L., J.L., B.Q., and R.W. performed the experiment and analyzed the data. F.L. wrote the manuscript.

## Declaration of interests

The authors declare no competing interests.

## STAR★Methods

### Key resources table

**Table:** 

REAGENT or RESOURCE	SOURCE	IDENTIFIER
**Software and algorithms**		

Windows 11	Microsoft	https://www.microsoft.com/windows/
MATLAB R2020a	MathWorks	https://www.mathworks.com/

**Other**		

Computer workstation	Dell	T3650

### Method details

#### Overall details of the real-time measurement process

To calculate the electron spin polarization $P_{z}^{e}\left(r\right)$ at the center of the cell using Equation 22, the value of the pump laser power P(r) at the cell’s center (which cannot be directly measured) is essential. As shown in our hardware setup, the DPU cannot directly obtain the pump laser power P(0) at the initial position inside the cell in real-time, and thus cannot use Equation 12 along with the transmitted laser power $P_{\text{trmt}}$ to compute this value. Therefore, we generated a large dataset using Equation 12 and trained a NN to estimate the pump laser power P(r) at the center of the cell based on the transmitted laser power $P_{\text{trmt}}$ and the cell temperature $T_{\text{cell}}$. Finally, the *in situ* real-time measurement of the electron spin polarization $P_{z}^{e}\left(r\right)$ is realized.

#### Pump laser propagation model

At room temperature, the laser power attenuation is primarily due to scattering by noble and quenching gases, while under heating conditions, additional attenuation occurs due to absorption by alkali atoms.

#### Parameter estimation based on the Euler-PSO algorithm

The Euler method approximately discretizes the differential equations governing the system for iterative computation. The PSO algorithm, inspired by the social behaviors of flocks and schools, optimizes parameters by simulating the movement of particles in a multi-dimensional space, eliminating the need for gradient-based information. The PSO adjusts the velocity and position of each particle based on both personal and global best solutions, with an adaptive inertia weight that strikes a balance between exploration and exploitation of the search space. The fitness function, which evaluates the closeness of the model output to experimental data, drives the optimization process. The proposed Euler-PSO algorithm is implemented in MATLAB R2020a. By combining experimental data, the algorithm identifies the unknown parameters of the established model.

#### Electron spin polarization measurement using Neural network

The NN training is conducted on a computer workstation with an Intel Core i9-11900K CPU. The cell temperature, $T_{\text{cell}}$, and the laser power at the end of the cell, P(2r), are used as output data, while the laser power at the center of the cell, P(r), serves as input data. The final electron spin polarization is determined by combining the trained NN output with the previously obtained parameter, providing an effective model for predicting electron spin polarization.

### Quantification and statistical analysis

All statistical analyses and results are described in the relevant sections. The data are generated using MATLAB R2020a based on the raw experimental data.
